# Good practices for ^89^Zr radiopharmaceutical production and quality control

**DOI:** 10.1186/s41181-024-00258-y

**Published:** 2024-05-11

**Authors:** Thomas Erik Wuensche, Serge Lyashchenko, Guus A. M. S. van Dongen, Danielle Vugts

**Affiliations:** 1 Department of Radiology & Nuclear Medicine, Amsterdam UMC Location Vrije Universiteit Amsterdam, De Boelelaan 1117, Amsterdam, The Netherlands; 2 Amsterdam Neuroscience, Brain Imaging, Amsterdam, The Netherlands; 3 Department of Radiology, Memorial Sloan Kettering Cancer Center, New York, USA

**Keywords:** Immuno-PET, Zirconium-89, DFO

## Abstract

**Background:**

During the previous two decades, PET imaging of biopharmaceuticals radiolabeled with zirconium-89 has become a consistent tool in preclinical and clinical drug development and patient selection, primarily due to its advantageous physical properties that allow straightforward radiolabeling of antibodies (^89^Zr-immuno-PET). The extended half-life of 78.4 h permits flexibility with respect to the logistics of tracer production, transportation, and imaging and allows imaging at later points in time. Additionally, its relatively low positron energy contributes to high-sensitivity, high-resolution PET imaging. Considering the growing interest in radiolabeling antibodies, antibody derivatives, and other compound classes with ^89^Zr in both clinical and pre-clinical settings, there is an urgent need to acquire valuable recommendations and guidelines towards standardization of labeling procedures.

**Main body:**

This review provides an overview of the key aspects of ^89^Zr-radiochemistry and radiopharmaceuticals. Production of ^89^Zr, conjugation with the mostly used chelators and radiolabeling strategies, and quality control of the radiolabeled products are described in detail, together with discussions about alternative options and critical steps, as well as recommendations for troubleshooting. Moreover, some historical background on ^89^Zr-immuno-PET, coordination chemistry of ^89^Zr, and future perspectives are provided. This review aims to serve as a quick-start guide for scientists new to the field of ^89^Zr-immuno-PET and to suggest approaches for harmonization and standardization of current procedures.

**Conclusion:**

The favorable PET imaging characteristics of ^89^Zr, its excellent availability due to relatively simple production and purification processes, and the development of suitable bifunctional chelators have led to the widespread use of ^89^Zr. The combination of antibodies and ^89^Zr, known as ^89^Zr-immuno-PET, has become a cornerstone in drug development and patient selection in recent years. Despite the advanced state of ^89^Zr-immuno-PET, new developments in chelator conjugation and radiolabeling procedures, application in novel compound classes, and improved PET scanner technology and quantification methods continue to reshape its landscape towards improving clinical outcomes.

## Background

**Fig. 1:**
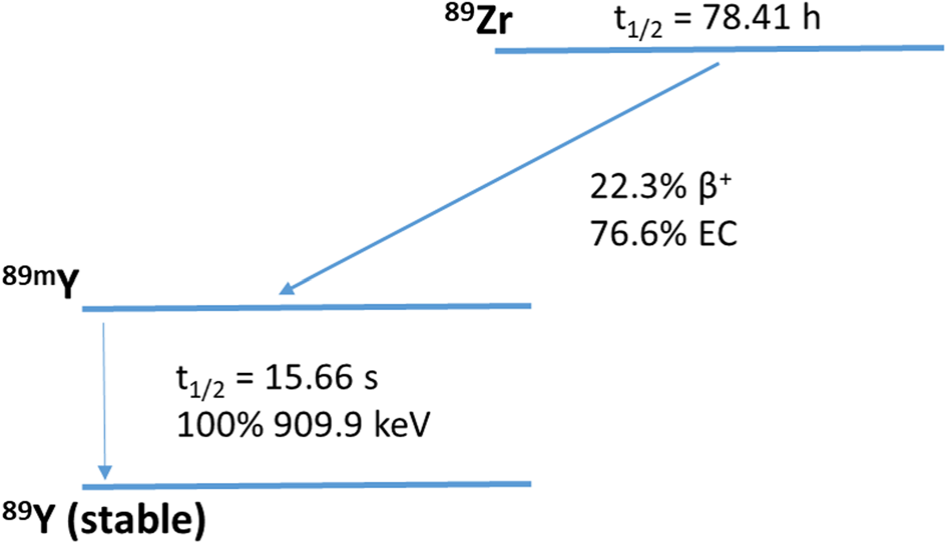
Simplified decay scheme of Zirconium-89

Although the production of zirconium-89 from yttrium-89 using a (p,n) reaction was reported in 1966 (Yamazaki et al. 1964) there was little to no interest in the radionuclide until 1990, when a purification procedure was reported (Dejesus and Nickles 1990). Hereafter, the developments followed up rapidly, which is mainly due to the recognition of ^89^Zr as a potential positron emission tomography (PET) radionuclide to radiolabel monoclonal antibodies (mAbs), an emerging class of therapeutics in various diseases up to this day (Chomet et al. 2021; Verel et al. 2003; Dongen et al. 2021). With a physical half-life of 3.3 days, ^89^Zr allows PET imaging of large molecules with slow kinetics like antibodies for up to a week with conventional scanners or, in the case of long axial field-of-view scanners, even up to 30 days (Dongen et al. 2021; Berg et al. 2020). Zirconium-89 decays via positron emission (22.3%) and electron capture (76.6%) to ^89m^Y, which decays via gamma-ray emission to stable yttrium-89 (Fig. 1). The photons (909 keV) from the metastable state do not overlap with the PET signal (511 keV) and decay around 16 s later. Due to the low energy of the ^89^Zr-positrons (E_β+,ave_ = 396.9 keV), the average travel range of the positrons is 1.2 mm and PET images can be obtained at high resolution (Beekman et al. 2021).

**Fig. 2:**
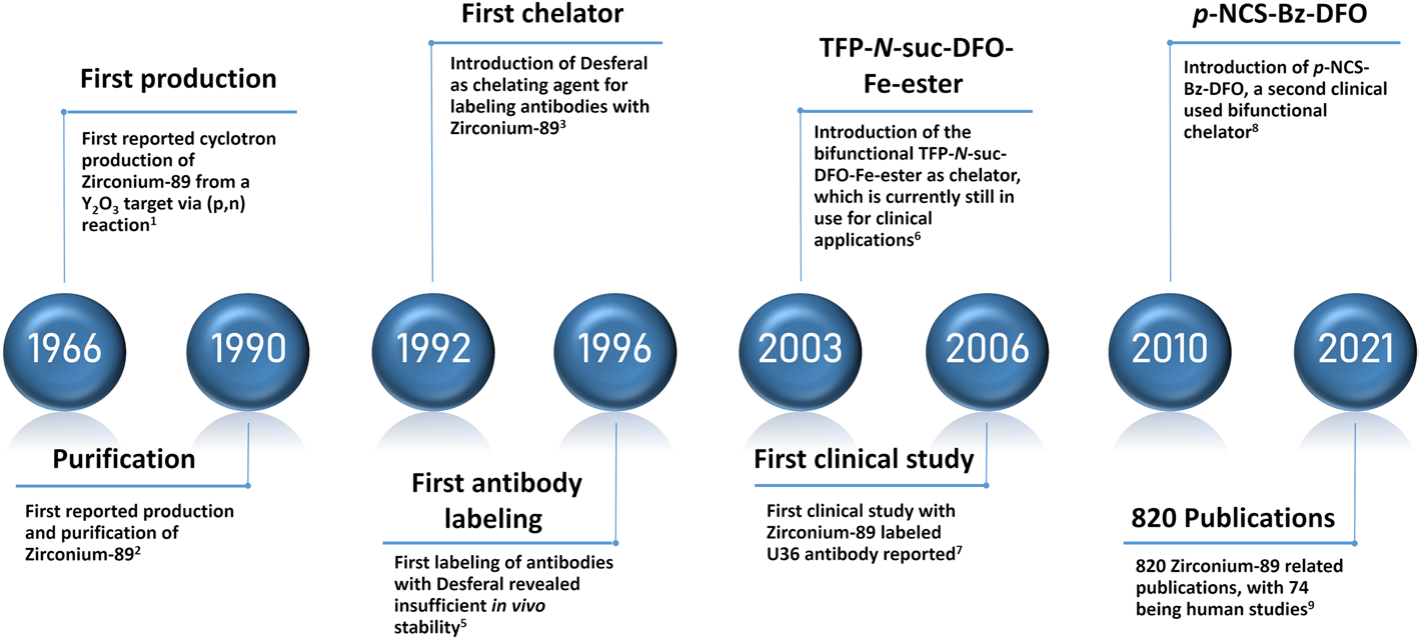
Timeline of Zirconium-89 in PET imaging highlighting the milestones

After the introduction of two of the most commonly used bifunctional chelators (Fe-DFO-*N*-suc-TFP ester and *p*-NCS-Bz-DFO) (Verel et al. 2003; Vosjan et al. 2010), interest in combining the high affinity and specificity of monoclonal antibodies with the favorable PET imaging properties of ^89^Zr (^89^Zr-immuno-PET) grew, resulting in an increasing number of publications. The first clinical study in 2006 was performed with an ^89^Zr-labeled monoclonal antibody for detecting lymph node metastases, U36, in head and neck cancer patients (Börjesson et al. 2006). Follow-up studies provided assessments of radiation doses, which appeared at the high end. Due to this limitation, the number of repeated applications of ^89^Zr-immuno-PET for one patient was restricted. However, this became less of a concern due to advancing PET scanner sensitivities (Dijkers et al. 2009; Börjesson et al. 2009). A thorough review by De Feo et al. (2022) estimated 820 ^89^Zr related articles, of which 74 were human studies, showcasing the rapidly grown interest in this radionuclide. A timeline with a selection of milestones is given in Fig. 2.

With the continuously growing interest in ^89^Zr to label antibodies and antibody derivatives for ^89^Zr-immuno-PET and its new applications to various other compound classes (e.g., living cells, nanoparticles and microplastics), there is a need to provide valuable recommendations and standards to the ^89^Zr community. This review will give an overview of key aspects in ^89^Zr-radiochemistry and radiopharmacy: production of ^89^Zr, chelator conjugation and radiolabeling of antibodies, and quality control of the resulting radiolabeled antibodies. This review aims to give a quick start for scientists newly entering the field while presenting approaches to harmonize current procedures.

## Routinely used chelators for ^89^Zr-immuno-PET

**Fig. 3:**
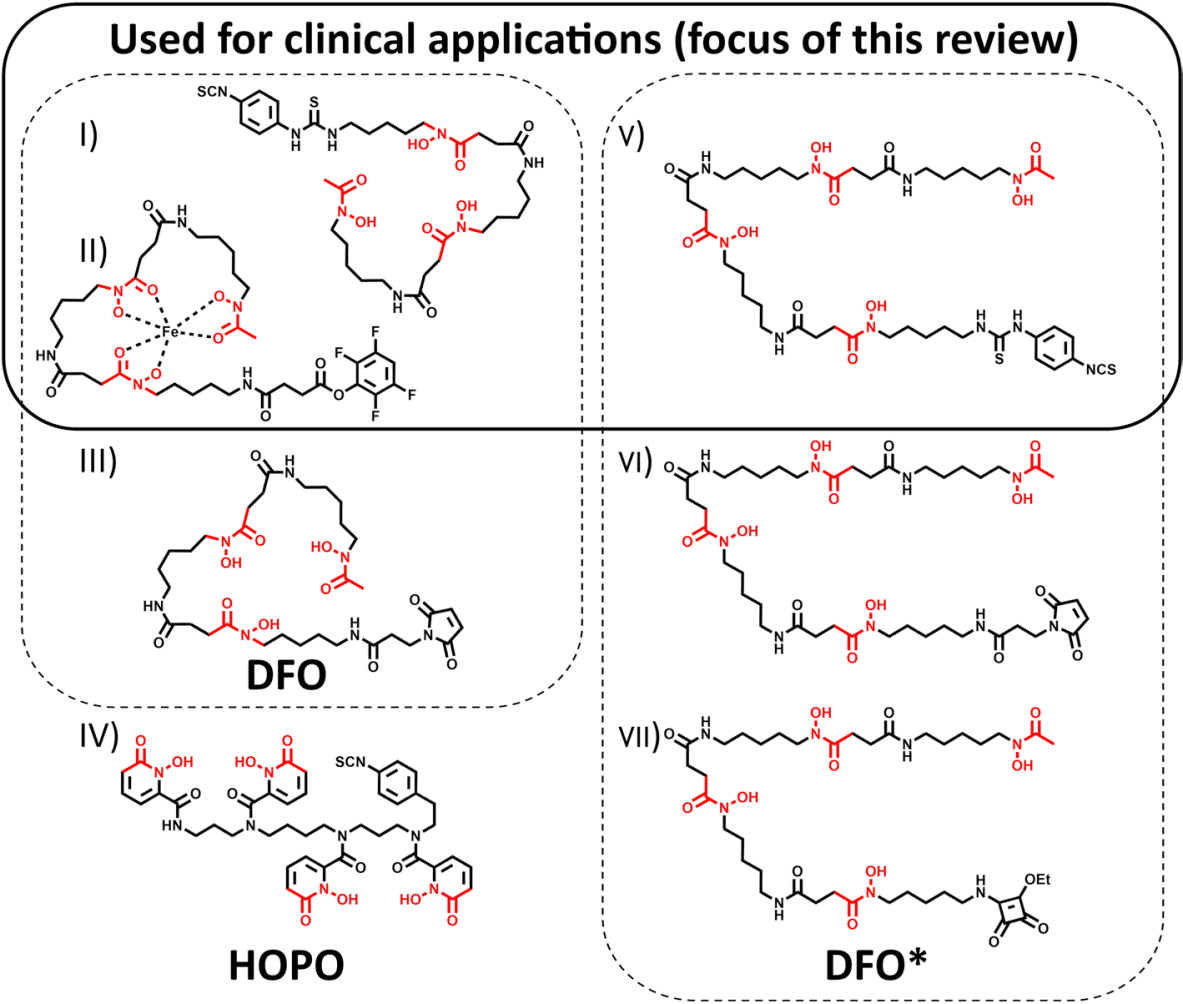
Structures of commercially available bifunctional chelators routinely used for ^89^Zr-immuno-PET. I) Fe-DFO-N-suc-TFP ester II) p-NCS-Bz-DFO III) Mal-DFO IV) p-NCS-Bz-HOPO V) p-NCS-Bz-DFO* VI) Mal-DFO* VII) Sq-DFO*

Zirconium-89 coordination chemistry is centered around its oxidation state + 4. The most commonly used bifunctional chelators until today are based on Desferrioxamine (DFO), also known as Deferoxamine (DFOA), its brand name, Desferal® (Df) or, in the case of the free base, Desferrioxamine B (DFOB) (Fig. 3). These hydroxamate-based chelators allow radiolabeling at room temperature in aqueous conditions with sufficient reaction times, considering the long half-life of ^89^Zr (30–60 min radiolabeling reaction time vs. 78.4 h half-life). Classical chelators like DOTA or DTPA derivates, which are used for a variety of radiometals (e.g.,^*^Y, ^111^In, ^64^Cu, ^177^Lu), have also been evaluated but are not or less suitable. DTPA-pBz-NCS, conjugated to Zevalin, showed poor radiolabeling yields (< 0.1%) (Perk et al. 2006). ^89^Zr-DOTA showed promising results in stability tests, but due to the need for elevated temperatures to form the radiometal-chelator complex, its application is limited to prelabeling procedures in the case of temperature-sensitive biologics (Heskamp et al. 2017). Also, no additional precautions related to the demetallization of reagents and materials, besides using high-purity grade chemicals, are needed when using DFO derivates. The DFO siderophore is an approved pharmaceutical clinically used for aluminum and iron demetallation therapies. This facilitated the translation of ^89^Zr-immuno-PET to the clinic due to its known safety profile and availability (Mobarra et al. 2016). Initial studies demonstrated promising in vitro stability of the Zr-DFO complex (Meijs et al. 1992). However, ^89^Zr-labeled proteins derived from the first published bifunctional variant, SATA-DFO, exhibited inadequate in vivo stability (Meijs et al. 1996). Until today, Fe-DFO-*N*-suc-TFP ester and *p*-NCS-Bz-DFO for lysine conjugation are the two most often used bifunctional chelators for ^89^Zr-immuno-PET in clinical set-ups, but also a maleimide version is commercially available for cysteine conjugation (Fig. 3) (Verel et al. 2003; Vosjan et al. 2010; Cohen et al. 2013; Zeglis and Lewis 2015; Deri et al. 2013). Nonetheless, with its three hydroxamate groups, the chelator provides six of the eight required donor atoms for Zr^4+^_,_ which leaves two binding sides open without firm coordination. Due to unwanted transmetallation in vivo, bone uptake is often observed in a preclinical setting. Although the deposition of ^89^Zr in bone has not been routinely observed in published clinical studies to date, a systematic review is required to evaluate this question further. A resolution to this problem is particularly important since non-specific bone uptake will increase the patient's radiation burden and may contribute to the misidentification of bone metastases (Chomet et al. 2021; Vugts et al. 2017). Therefore, a considerable effort from several groups in the past years led to various novel chelator designs, providing eight donor atoms for potentially higher in vivo stability (Feiner et al. 2021a). Of all these new candidates, two chelator groups are used routinely. DFO* (or DFOstar) is a DFO derivate carrying one additional hydroxamate group. Three bifunctional versions (Mal-DFO*, *p*-NCS-Bz-DFO*, and sq-DFO*) are commercially available, of which *p*-NCS-Bz-DFO* is currently under clinical investigation (89Zr-DFO* 2023). Another bifunctional chelator, HOPO, based on hydroxypyridinone groups, is commercially available but has not yet been used in clinical applications. Notably, when considering clinical applications with novel chelator scaffolds, one has to be mindful of the potential time and costs associated with extensive cytotoxicity studies that would be required. An overview of those routinely used bifunctional chelators is given in Fig. 3; examples of the Zr-complexes’ DFT-optimized structure are reported (Patra et al. 2014; Deri et al. 2015; Holland 2020). The three chelators that are commercially available and used for clinical applications are the selected chelators of this review.

## Production of Zirconium-89

An advantage of ^89^Zr, compared to other radiometal PET emitters, is the practically scalable radionuclide availability. The production and radiochemical isolation methodology of ^89^Zr is well-established, relatively simple, and comparatively inexpensive. In fact, several bespoke solid targets and single-use cassette-based purification systems have become commercially available in recent years that provide a turn-key solution for in-house ^89^Zr production in facilities already equipped with biomedical cyclotrons. In addition, several commercial suppliers make ^89^Zr worldwide available at a reasonable price.

One facilitating factor that makes ^89^Zr production straightforward is the ability to produce it in industrial-level quantities on medical cyclotrons using the simple ^89^Y(p,n)^89^Zr reaction (Gaja et al. 2020; Queern et al. 2017). The target material ^89^Y is 100% naturally abundant, making it an ideal target material because it is inexpensive, does not require recycling, and does not yield radionuclidic impurities from isotopic impurities present in the target material. However, despite the 100% abundance, the one key consideration relevant to ^89^Y target irradiation is to keep the proton energy below 13 MeV in order to avoid the formation of ^88^Zr radioimpurity via the ^89^Y(p,2n)^88^Zr reaction, which has an 83.4 day radioactive half-life and cannot be separated from the radioisotope of interest during subsequent radiochemical isolation (Infantino et al. 2011).

Production of ^89^Zr using liquid cyclotron targetry systems has been reported (Pandey et al. 2014). However, the comparatively limited quantities of ^89^Zr that can be produced using liquid targets have positioned the solid targets as the preferred production method. The 100% enriched solid target material is available from various suppliers, usually in the form of either yttrium foil or a thin layer of yttrium that has been sputtered onto niobium backing (Vugts et al. 2017). These targets are typically irradiated at 40–45 µA for 2–4 h, yielding > 1110 MBq at EOI (end-of-irradiation) (Verel et al. 2003; Gaja et al. 2020; Queern et al. 2017).

Following the ^89^Y target irradiation, the obtained ^89^Zr has to be radiochemically isolated to yield the ^89^Zr precursor solution. The most commonly applied ^89^Zr purification procedure is based on the methodology reported initially by Verel et al. and involves dissolving the target in concentrated HCl, and loading it onto hydroxamate resin, trapping the ^89^Zr onto the resin, washing the hydroxamate resin with 2 M HCl and water to eliminate the residual yttrium, and eluting the ^89^Zr from the hydroxamate resin with 1 M oxalic acid solution (Verel et al. 2003; Queern et al. 2017). Lower concentrations of oxalic acid (down to 0.05 M) or other reagents like sodium citrate have been reported for elution, potentially improving subsequent radiolabeling applications (Wichmann et al. 2023; Larenkov et al. 2019). This process effectively removes elemental impurities, including trace radionuclidic impurities that may have been formed from the irradation of target components (Gaja et al. 2020).

The requirements for characterizing the batches of ^89^Zr produced vary by region and depend on how the material is further processed during the subsequent radiopharmaceutical manufacture. In all instances, periodic evaluation of trace radionuclidic impurities and confirmation of radionuclidic identity on every produced batch is generally recommended. Other applied acceptance specifications may also include determining the trace metals content and specific activity. However, the applicability of these specifications may not be relevant in situations when high-quality chemicals and reagents with the least amount of metal impurities are used during production or when adequate radio-incorporation is consistently achieved due to significant access of a particular chelator used in the radiopharmaceutical manufacturing process, or when the material is subjected to additional purification during radiosynthesis.

In summary, the favorable naturally occurring radionuclide characteristics of ^89^Zr, combined with the relative simplicity of both ^89^Zr solid target irradiation and subsequent radiochemical purification, have led to ^89^Zr PET overcoming one of the most commonly encountered problems in radiopharmaceutical clinical development—availability.

## Chelator conjugation of antibodies and subsequent radiolabeling with ^89^Zr

Methods in which the antibody is first conjugated with the bifunctional chelator, followed by radiolabeling with ^89^Zr, are most widely applied. Pre-labeling approaches, radiolabeling the bifunctional chelator first and subsequent conjugation to the antibody, are not favored since they lead to lower radiochemical yields and contradict the ALARA (as low as reasonably achievable) radiation safety principle due to the more prolonged radiolabeling procedures. Therefore, they are rare and limited to distinct applications like labeling of living cells and, thus, will not be further discussed. The following section is based on standardized protocols for regular mAbs (commercially availably IgG1 for human use; M_W_ ~ 150 kDa) and overviews of commonly experienced issues. Since complications can arise before the start of radiolabeling, when the chelator conjugation is performed, these are also discussed in detail, along with strategies for troubleshooting. A summary is given in Table 1.

**Table 1:** Commonly encountered problems in mAb chelator conjugation and troubleshooting

Step	Problem	Possible reason	Solution
pH adjustment for the conjugation reaction	Large volumes of base needed to adjust pH to 9–9.5	Antibody in strong storage buffer	Buffer exchange
Conjugation reaction with Fe-DFO-*N*-suc-TFP ester	Impaired mAb integrity Low antibody recovery	Low pH during Fe removal	Use *p*-NCS-Bz-DFO/DFO* as alternative
Conjugation reaction with *p*-NCS-Bz-DFO/DFO*		Higher lipophilicity and reactivity of *p*-NCS-Bz-DFO/DFO*	Change the addition order of chelator to mAb (see *p*-NCS-Bz-DFO^(*)^conjugation of mAbs) Lowering conjugation temperature to ambient temperature or 4°C
Conjugation reaction with all chelators	Low conjugation efficiency	Buffer affects the conjugation efficiency	Buffer exchange
Purification after chelator conjugation	Residues of free chelator in mAb fraction	Larger amounts of bifunctional chelator, as described in standard procedures, are used	Additional purification step
	Impaired mAb integrity	Spin filtration used for purification	Use Less harsh methods (e.g., desalting column)
Determination of chelator-to-mAb ratio when *p*-NCS-Bz-DFO/DFO* is used	Inaccurate results with LC–MS-based methods	Low signal strength due to too many thiourea moieties, high mass of antibody, or both	Alternative methods like titration (see assessment of chelator-to-antibody ratio) Reduction of disulfide bonds to analyze HC and LC separately
Assessment of chelator-to-mAb ratio	Too low ratio (after having tested buffer exchange)	Active group (NCS or TFP) hydrolyzed	Use dry solvents and store aliquots under inert gas Consider condensing water when using batches stored at low temperatures
		Higher amounts are needed as in standardized procedures	Higher amounts of bifunctional chelator Lower total reaction volume Longer reaction times

### Fe-DFO-*N*-suc-TFP ester conjugation

**Fig. 4:**
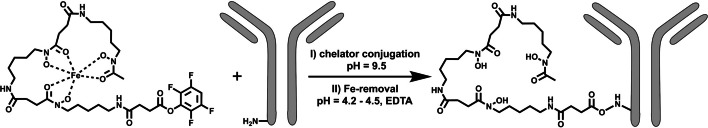
Chelator conjugation with Fe-DFO-N-suc-TFP ester and subsequent Fe-removal

Fe-DFO-*N*-suc-TFP ester can be obtained via known synthesis procedures or commercial sources. The following section is based on standard step-by-step operating procedures and protocols for regular mAbs (commercially availably IgG1; M_W_ ~ 150 kDa) (Verel et al. 2003). Fe-DFO-*N*-suc-ester is a brown solid soluble in acetonitrile, and aliquots of the chelator in MeCN can be stored at -70 °C for at least a year. Chelator conjugation is done at pH ~ 9.0–9.5. For most antibodies, the formulation conditions allow direct pH adjustment using a dilute inorganic base like 0.1 M Na_2_CO_3_ to adjust the pH (Fig. 4). For some antibodies, buffer exchange before pH adjustment may be mandatory for two reasons: 1. To remove formulation components with primary amines (TRIS or glycine-containing buffers) or other components that could cause a lower conjugation efficiency; 2. To remove highly formulation components that can lead to the need for excessive amounts of base for pH adjustment. A buffer exchange to saline for injection (0.9% NaCl) with subsequent pH adjustment or with a carbonate buffer at the required pH is recommended.

**Fig. 5:**
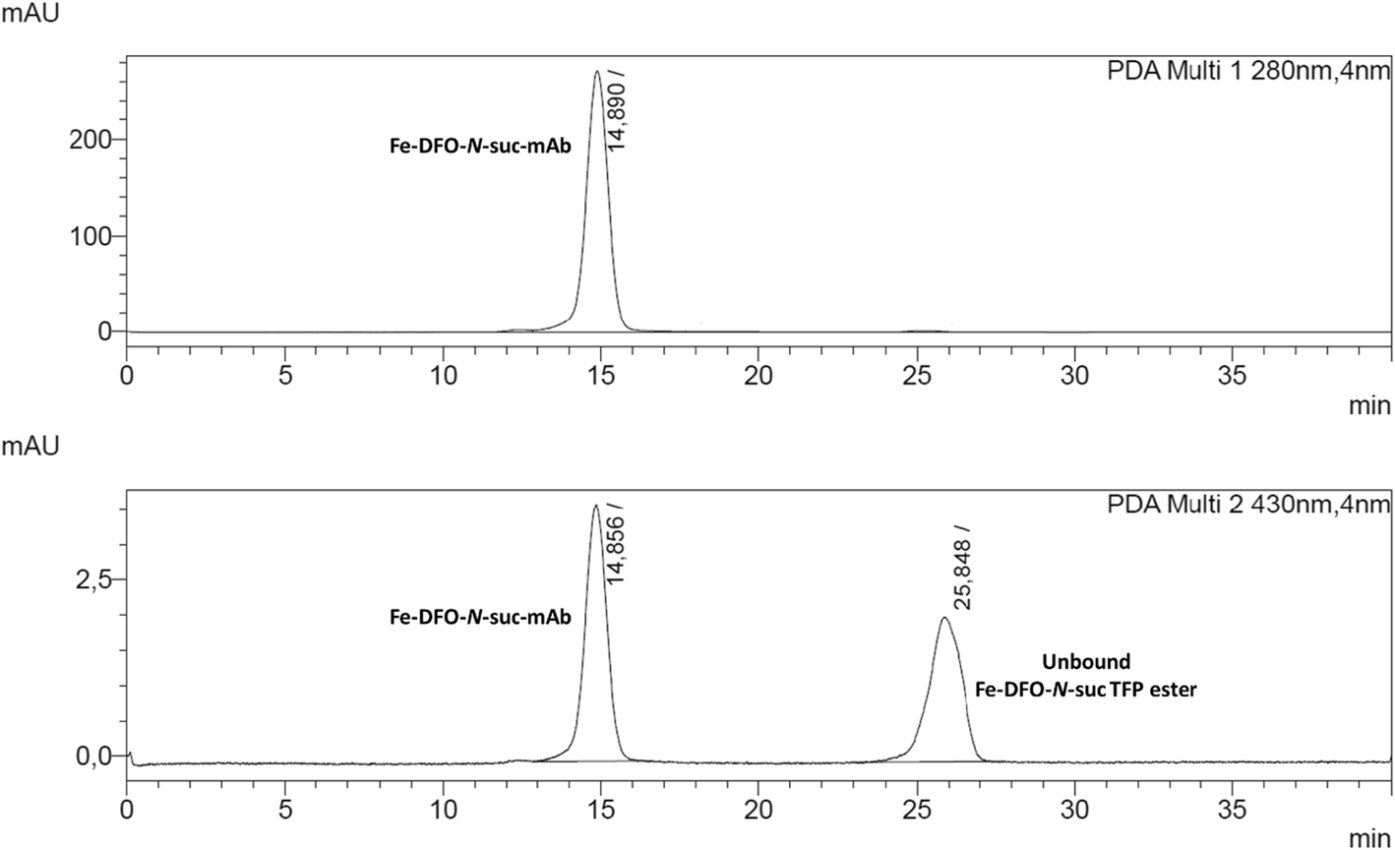
SE-HPLC chromatogram (wavelength at 280 nm and 430 nm) of a Fe-DFO-N-suc-TFP ester chelator conjugated mAb before the subsequent Fe-removal and purification. The ratio between the Fe-DFO-N-suc-mAb and unbound Fe-DFO-N-suc TFP ester can be used to determine the chelator-to-mAb ratio

With the usual two molar equivalents of chelator in MeCN, around 2 vol% of MeCN are present in the reaction mixture. Using the standard 5 mg/mL antibody concentration for conjugation results in conjugates with chelator-to-mAb ratios ranging from 1 to 1.5. If more molar equivalents are needed and more MeCN has to be added, caution has to be taken concerning antibody integrity (percentage of antibody monomer). Usually, after initial mixing using a pipette or gentle mixing by hand, 30 min of reaction time at room temperature without further mixing is sufficient at pH ~ 9.0–9.5. Lower pH values (8.5–9.0) can be used for sensitive antibodies but will most certainly lead to longer reaction times to reach identical chelator-to-mAb ratios. Before Fe-removal, a small aliquot of the reaction mixture can be taken to determine the chelator-to-mAb ratio via SE-HPLC measurement at 430 nm by comparing the amount coupled to the antibody and the amount of unreacted/hydrolyzed Fe-DFO-*N*-suc-species (Fig. 5). After conjugation, the Fe, which protects the hydroxamate groups, must be removed to allow ^89^Zr-radiolabeling. The protection via Fe is needed since otherwise, the activated TFP-ester (and any activated ester) reacts with the hydroxamate groups of DFO. Fe-removal is typically done using an excess of EDTA at low pH (pH 4.2–4.5) during a 30-min incubation at 37 °C. For controlled pH adjustment, a weaker acid like gentisic acid (2,5-hydroxybenzoic acid) in larger portions is initially used, followed by a strong, dilute acid like 0.25 M H_2_SO_4_ in very small amounts, mixing after each addition. This step must be performed cautiously since a drop below pH 4.2 will likely affect the antibody functionality and integrity. After iron removal, the color of the solution should become colorless, indicating the removal of Fe from DFO and transchelation to EDTA (Verel et al. 2003).

When this method was established, the efficiency of the Fe-removal had to be tracked precisely. Using the change of UV–Vis absorption at 430 nm of the product during SE-HPLC analysis and thereby monitoring the Fe-removal is hampered by the product complexing Fe released from the HPLC system. A delicate metal-free set-up would be required to obtain reliable information on the Fe-removal efficiency. Therefore, [^59^Fe]Fe-DFO-*N*-suc-TFP ester was used to track the Fe-removal via a radiodetector. Notably, highly sensitive methods like ICP-MS could be a viable alternative to determine the amount of Fe left after purification.

**Critical step:** The Fe-removal at low pH can result in precipitates and formation of soluble non-covalent aggregates (impaired antibody integrity). Determining the antibody integrity and recovery (see quality controls) is crucial before starting the radiolabeling step. If the antibody cannot withstand these relatively harsh conditions, conjugation with *p*-NCS-Bz-DFO or *p*-NCS-Bz-DFO* is a viable alternative.

### *p*-NCS-Bz-DFO and *p*-NCS-Bz-DFO* conjugation of antibodies

**Fig. 6:**
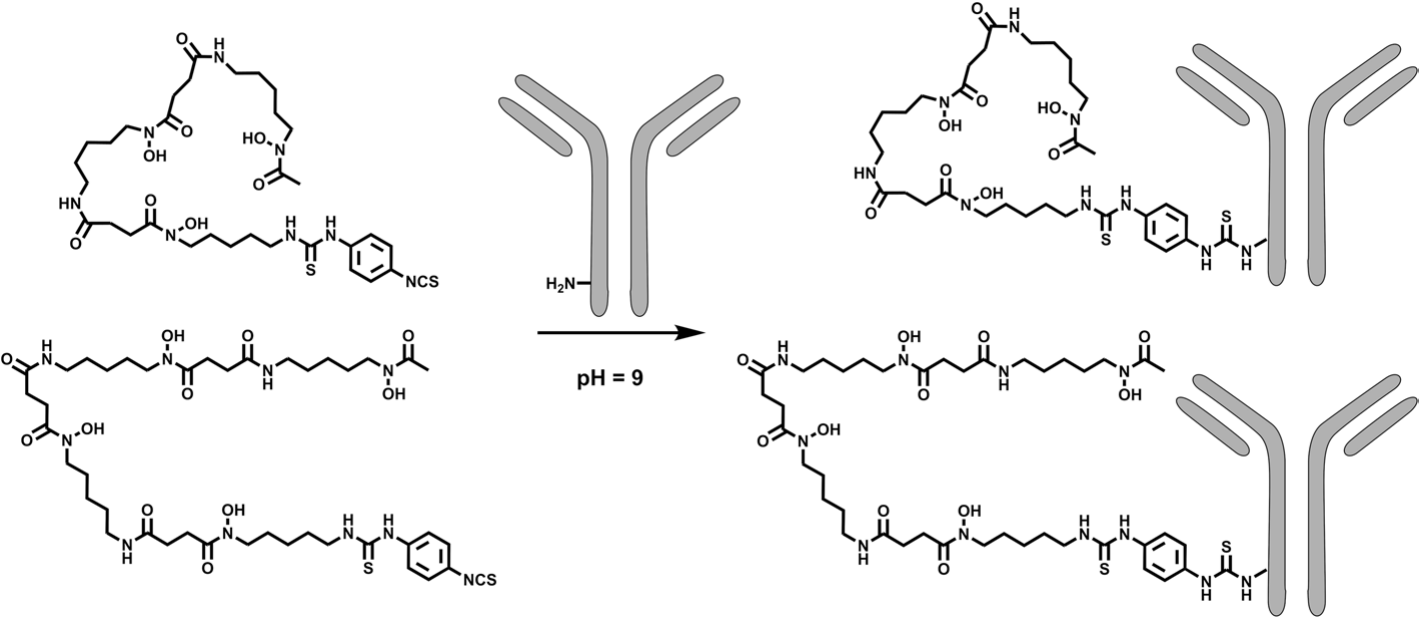
Chelator conjugation with p-NCS-Bz-DFO and p-NCS-Bz-DFO*

*p*-NCS-Bz-DFO and *p*-NCS-Bz-DFO* (also called *p*-NCS-Bz-DFOstar) can be obtained via known synthesis procedures or commercial sources. The following section is based on standard step-by-step procedures and protocols for regular mAbs (commercially availably IgG1 for human use; M_W_ ~ 150 kDa) (Chomet et al. 2021; Cohen et al. 2013; Deri et al. 2013; Sharma et al. 2021). Procedures for chelator conjugation with *p*-NCS-Bz-DFO and *p*-NCS-Bz-DFO* are very similar and will be discussed together in this section. Usually, chelator aliquots in DMSO are used, which can be stored in the freezer for up to a year. Usually, three molar equivalents of chelator and a 5 mg/mL antibody solution lead to conjugates with a chelator-to-mAb ratio between 1 and 1.5. For the conjugation of antibodies with *p*-NCS-Bz-DFO/DFO*, the protection of the hydroxamate groups is unnecessary since the isothiocyanate is not reactive towards hydroxamates, allowing a one-step chelator conjugation. The conjugation is usually done at a pH ~ 9 at 37 °C (Fig. 6). As described before, the pH adjustment and optional buffer exchange are similar to the Fe-DFO-*N*-suc-TFP ester conjugation*.* Due to the lower solubility of *p*-NCS-Bz-DFO/DFO*, DMSO is typically used to dissolve the bifunctional chelator, and 2–5 vol% are used in the conjugation. In comparison with the Fe-DFO-*N*-suc-TFP ester, the addition of the *p*-NCS-Bz-DFO/DFO* in DMSO to the antibody has to be performed with more precautions to avoid precipitations and formation of soluble high molecular weight aggregates. Several options are possible to prevent or reduce unwanted impairment of the antibody integrity:

A. The *p*-NCS-Bz-DFO/DFO* in DMSO is added in small portions to the antibody solution, which was set to pH ~ 9 beforehand. Immediate and repetitive mixing with the pipette is performed to ensure thorough mixing of the reaction solution. If soluble high molecular weight aggregates are formed, is highly dependent on the operator's pipetting skills.
B. The antibody solution at pH ~ 9 is added to the small volume of *p*-NCS-Bz-DFO/DFO* in DMSO, and immediate repetitive pipetting is performed to ensure proper mixing.
C. The chelator in DMSO is mixed with a small amount (100–200 µL) of buffer in advance and added portion-wise, mixing after each addition, to the antibody solution. The pH adjustment takes place after the complete stepwise addition of the chelator. Caution is advised when this method is applied to *p*-NCS-Bz-DFO* due to its tendency to induce precipitation.

It is crucial to determine the antibody integrity and recovery (see quality controls) before radiolabeling.

### Purification of DFO/DFO*-conjugated antibodies

The DFO/DFO*-conjugated antibodies can be purified via simple size exclusion methods, e.g., desalting columns and spin-filtration (gravity flow and spin, respectively), with suitable size thresholds for the corresponding antibody. Notably, spin-filtration methods are considered harsher than methods like desalting columns due to the risk of over-concentration. For the subsequent radiolabeling, determination of the exact antibody concentration is recommended, which can be done using SE-HPLC or nanodrop UV spectrophotometer. The exact concentration can be calculated based on a calibration curve or via the molar extinction coefficient (based on the antibodies amino acid sequence). A calibration curve is usually preferred over the use of the extinction coefficient. The latter is faster but less accurate and has a higher limit of detection (LoD—the lowest analyte concentration likely to be reliably distinguished from blank samples; also referred to as lower limit of detection or minimum detectable concentration) and limit of quantification (LoQ—the minimum concentration at which the analyte can be consistently detected and where specific predefined criteria for accuracy and precision are achieved; also referred to as lower limit of determination or lower end of measuring range) (NCCLS 2008; Armbruster and Pry 2008). Methods like the Bradford antibody assay are less common in routine set-ups. If a higher antibody concentration is required in the radiolabeling, an additional spin-filtration to concentrate may be performed. Determining the antibody integrity (see quality controls) is recommended if a concentration step is performed. If the radiolabeling is performed within a short time period after the chelator conjugation, elution with 0.9% NaCl or 0.5 M HEPES and storage at 2–8 °C is often sufficient.

While unwanted antibody modifications (deamidation, oxidation and isomerization) are less of a concern in the relatively short periods of antibody conjugation, this can differ for longer storage times (Lu et al. 2019; Gupta et al. 2022). Therefore, conditions may vary, and other buffers (e.g., acetate, acetate/sucrose, or histidine/sucrose in a pH range of 5–6) and lower storage temperatures (− 20 °C or − 80 °C) may be preferred. For commercial antibodies, the initial storage buffer and temperature can be used for orientation. Buffers containing citrate must be avoided since they can reduce Zr^4+^ to Zr^2+^. The same accounts for phosphate buffered saline (PBS) due to the high affinity of phosphate to Zr^4+^, leading to higher rates of demetallation. Screening long-term storage conditions (buffer, temperature, freeze–thaw circles) is time-intensive. However, it can still be beneficial, especially for batch-wise clinical productions, since one conjugation can be used for up to several years for immediate radiolabeling on the day of radiotracer production.

### Assessment of chelator-to-antibody ratio

Standardized procedures with regular mAbs for regular mAbs (commercially availably IgG1 for human use; M_W_ ~ 150 kDa) at 5 mg/mL, using 2 eq. of Fe-DFO-*N*-suc-TFP ester or 3 eq. *p*-NCS-Bz-DFO/DFO*, lead to chelator-to-mAb ratios between 1 and 1.5 (Verel et al. 2003; Vosjan et al. 2010). Nevertheless, if a facility establishes zirconium-89 radiochemistry, monitoring the efficiency in the initial conjugations is recommended since this can be beneficial for clinical application and possible troubleshooting if radiolabeling yields turn out to be not high enough. Furthermore, the number of equivalents of bifunctional chelator necessary can differ if other molecules than mAbs are used, if higher ratios of chelator-to-mAb ratio are needed to achieve higher molar activities in the subsequent radiolabeling or if the concentration of antibody is lower. As mentioned in the section on mAb chelator conjugation with Fe-DFO-*N*-suc-TFP ester, determining the chelator-to-mAb ratio is easily possible by performing a SE-HPLC analysis after chelator conjugation and before Fe-removal (Fig. 5). For *p*-NCS-Bz-DFO/DFO*, standard methods for assessment of chelator-to-mAb ratios apply. The most commonly used are mass spectrometry (LC–ESI–MS or MALDI-TOF) methods, with the advantage that relatively small amounts of antibody are required due to the high sensitivity of these methods (Feiner et al. 2021b). Sample preparation time, however, can be extensive since deglycosylation, buffer exchange, and salt removal may be necessary in the case of MALDI-TOF. In addition, when higher *p*-NCS-Bz-DFO/DFO*-to-mAb ratios are reached, some MS methods can become inaccurate or impaired (Chomet et al. 2021; Wuensche et al. 2022). An alternative to MS methods is a titration with carrier-added [^89^Zr]Zr-oxalate (known amounts of non-radioactive Zr-oxalate with trace amounts of [^89^Zr]Zr-oxalate) (Vugts et al. 2017). While it may have benefits, as no high-end equipment is needed, this method has the drawback of requiring larger amounts of antibody. This can be problematic if non-commercial antibodies are used. An even more straightforward approach, but with less accuracy than the titration, is to perform a *p*-NCS-Bz-DFO/DFO* conjugation without purification and immediately do a subsequent radiolabeling without purification (Chomet et al. 2021). This method assumes that radiolabeling of conjugated and free chelator is equally efficient.

### ^89^Zr-labeling of DFO/DFO*-conjugated antibodies

**Fig. 7:**
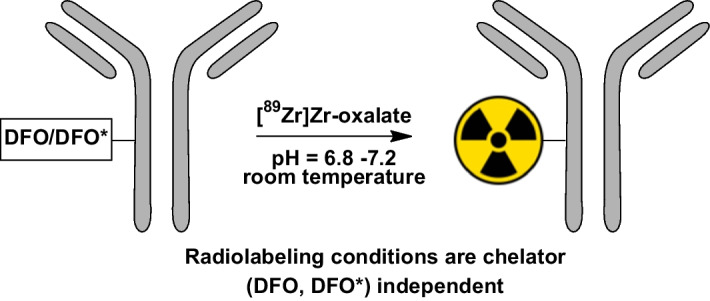
General radiolabeling procedure of DFO/DFO*-conjugated antibodies

The radiolabeling and subsequent purification conditions are similar for DFO-*N*-suc- and DFO/DFO*-*p*-Bz-NCS-conjugated antibodies (Verel et al. 2003; Vosjan et al. 2010; Zeglis and Lewis 2015; Deri et al. 2013; Vugts et al. 2017). Radiolabeling of hydroxamate chelators is preferably performed with [^89^Zr]Zr-oxalate in 1 M oxalic acid instead of [^89^Zr]ZrCl_4_ due to the higher stability towards hydrolysis. Several protocols describe similar reaction conditions at room temperature within a pH range of 6.8–7.2, using HEPES buffer between 0.25 and 1M and 2M Na_2_CO_3_ to adjust the pH before mixing it with the conjugated antibody (Fig. 7). The use of [^89^Zr]Zr-oxalate in dilute 0.05 M oxalic acid has also been reported, significantly reducing the amount of Na_2_CO_3_ (Wichmann et al. 2023). Nevertheless, a lower amount of carbonate ions can lead to more unsoluble condensated hydroxo species, potentially leading to a loss of radioactivity in the neutralization step (McInnes et al. 2017). The use of HEPES can be omitted if the pH of the reaction mixture is carefully checked. Alternatively, 1 M ammonium acetate solution can be used as a buffering system as it buffers the entire reaction mixture pH to 7.0, which is ideal for radioincorporation. Furthermore, it was recently shown that using 20 mM succinate buffers can improve radiolabeling efficiency and reduce reaction times (Wichmann et al. 2023). After the radiolabeling at room temperature under gentle shaking, a gravity size-exclusion column purification (e.g., PD-10) is most often used to purify the radiolabeled mAb from the reaction mixture. Also, other disposable size-exclusion columns can be used (e.g., HiTrap™ or HiPrep™, the latter in case of large-volume radiolabeling mixtures). Some protocols suggested an optional stripping of unreacted [^89^Zr]Zr^4+^ with DTPA before purification in the past, but this practice has become obsolete. A summary of commonly encountered problems and troubleshooting with radiolabeling and radioimmunoconjugates is given in Table 2.

**Table 2:** Commonly encountered problems and troubleshooting with radiolabeling and radioimmunoconjugates

Step	Problem	Possible reason	Solution
Neutralizing oxalic acid	Precipitation	Precipitation of sodium oxalate/oxalic acid	1. Adding HEPES before adding the antibody and mixing 2. If precipitation is still present: Carefully transfer supernatant without precipitants to the new reaction vessel If precipitation is a reoccurring problem: Shorter times between the addition of sodium carbonate and HEPES Check molarity of solutions used When ^89^Zr is formulated in 1 M oxalic acid and needs to be neutralized with 2 M sodium carbonate, the volume of sodium carbonate that is added prior to addition of either HEPES or 1 M Ammonium acetate should not exceed 45% of the volume of the radionuclide
Purification	Low radiochemical yield	Low chelator-to-mAb ratio	See Table 1
		Residues of free chelator	
		Reaction conditions	Longer reaction times Lower total reaction volume Check pH values and adjust to the correct pH value Use of 20 mM succinate buffer as an alternative to HEPES
Purification	Low radiochemical purity after having checked the chelator conjugation (see Table 1)	Low yield with related high amount of free Zr^4+^ species	
Storage till administration	Instability: Decreasing radiochemical purity Reduced immunoreactivity	Radiolysis	Avoid Cl^−^ containing formulations for *p*-NCS-Bz-DFO/DFO* Add anti-oxidants like L-methionine and *N*-acetyl cysteine (Avoid ascorbic acid) If possible: use Fe-DFO-*N*-suc-TFP ester instead of *p*-NCS-Bz-DFO/DFO*

### Formulation and final filtration

For clinical applications, it is essential to perform sterile filtration after the product is formulated in a suitable buffer for injection. Since the concentration of the ^89^Zr-labeled product can be very low and certain mAbs can also display lower hydrophilicity, checking tubes and containers used for sterile filtration and containers for long-term storage and transport for stickiness is essential. In addition to material compatibility considerations (glass or plastics, especially low-protein binding ones), small amounts of surfactants like Tween-20 or Tween-80 (0.05–0.1%) can be added to the buffer formulation to prevent the stickiness of the radiolabeled product. Furthermore, the addition of unlabeled antibody to the final formulation can also prevent disproportional loss of radioconjugate due to stickiness (See Quality controls – specific activity and antibody concentration). Regarding the choice of formulation buffer, the initial storage buffer of the mAb can be used for orientation. However, additional considerations are needed regarding radiolysis sensitivity and the properties of Zr^4+^. [^89^Zr]Zr-DFO-*N*-suc-mAbs can be formulated in various buffers, including saline, sodium acetate, and histidine. In contrast, [^89^Zr]Zr-DFO/DFO*-*p*-Bz-NCS-mAbs can not be stored in buffers containing Cl^−^, such as saline for injection (0.9% NaCl). Exposure to radiation leads to the radiolysis of water molecules, forming OCl^−^ if Cl^−^ is present. These ions exhibit a highly specific reactivity with the SH-group of the enolized thiourea unit. Citrate and phosphate-containing buffers should be avoided for all chelator counts since they cause instability of the ^89^Zr-labeled products. L-methionine and *N*-acetyl cysteine are suitable anti-oxidants to prevent radiolysis. In contrast, ascorbic acid and similar compounds that can function as a reducing agent should never be used since they will reduce Zr^4+^ to Zr^2+^, and ^89^Zr^2+^ will dissociate from the chelator.

## Quality controls

Generally, the same quality controls (QCs) as for other radioimmunoconjugates (e.g., ^177^Lu, ^111^In, ^64^Cu) must be performed. The extent of the QCs depends on whether the radioimmunconjugate is produced for research in a preclinical setting, a first-time validation of a new radioimmunoconjugate for clinical applications, or for routine clinical settings. This chapter discusses all relevant properties with their affiliated methods and equipment. An example of a routine test panel with required specifications for an ^89^Zr-labeled mAb to be i.v. administered in a volume of 10 or 20 mL to a patient is given in Table 3, and details are described subsequently.

**Table 3:** Example specification of an ^89^Zr-labeled mAb

Test	Specifications	Method
Appearance	Clear, colorless solution	Visual
pH product in histidine-sucrose-L-methionine-Tween-20	5.4–6.0	pH measurement using a pH meter or pH paper
Radiochemical purity	≥ 90.0%	Spin filter analysis^a^, iTLC^b^ or HPLC^c^
Antibody concentration	0.08–0.12 mg/mL	SE-HPLC UV at 280 nm
Antibody integrity	≥ 90.0% monomer	SE-HPLC^d^
Integrity filter	Bubble point value sterile filtration filter ≥ 3.2 bar	Pressure-hold test
Bacterial endotoxin analysis	≤ 2.5 EU/mL	Bacterial endotoxin tests (BET)
Antigen binding	≥ 70.0%^e^	Radio binding assay
Antibody identity	Binding to antigen confirmed	Radio binding assay
Labeling Yield	For information only	Spin filter analysis^a^, iTLC^b^, or HPLC^c^ or calculated based on purification
Sterility	No growth	Media fill^f^ or direct sterility testing of the final product

^a^Prefered for *p*-NCS-Bz-DFO/DFO*

^b^Suitable for Fe-DFO-*N*-suc-TFP ester

^c^Suitable for all chelators, but the assessment of HPLC recovery yield is mandatory

^d^SDS-PAGE is not a suitable alternative

^e^Maximum binding value differs depending on antibody/antigen combination

^f^Media fills may not be allowed, e.g., in the US, depending on applicable regulatory guidelines

### pH

pH measurements of an aliquot can be performed using a pH electrode or pH paper. Aside from pH measurement of the final product, using an aliquot after purification, in-process pH measurements should be performed throughout all critical steps of the procedure (e.g., after adding chelator, at the end of incubation steps) when implementing new procedures.

### Radionuclide identity, purity, and specific activity

-See radionuclide production-

### Radiochemical purity and stability

**Fig. 8:**
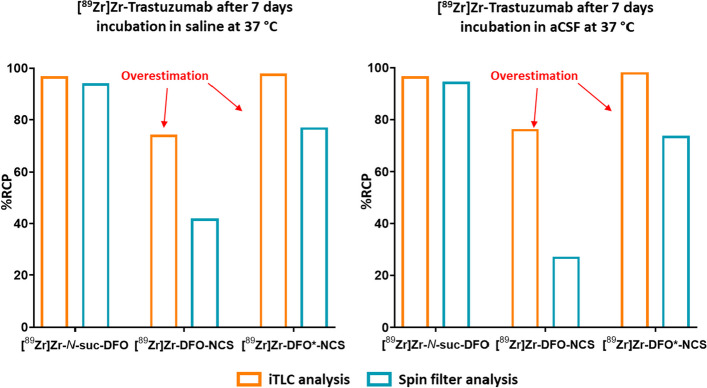
Example of stability data of ^89^Zr-labeled DFO-N-suc-mAbs and DFO/DFO*-p-Bz-NCS-mAbs in saline and artificial cerebrospinal fluid (aCSF) after 7 days. Radioimmunoconjugates were analyzed via iTLC (silica gel-impregnated glass fiber sheets; 20 mM citrate buffer pH = 5 + 55 mM EDTA, + 10 Vol% acetonitrile) and spin filtration (30 kDa cut-off spin filter; 50 mM sodium acetate/200 mM sucrose + 0.01% Tween-20, pH = 5.4–5.6 + 5 Vol% DMSO as wash buffer). These data demonstrate an overestimation of the radiochemical purity for the more lipophilic ^89^Zr-labeled DFO/DFO*-p-Bz-NCS-mAbs when iTLC analysis is used. In addition, these results showcase the superior stability towards radiolysis of [^89^Zr]Zr-DFO-N-suc-mAbs if no anti-oxidants are added

Determination of radiochemical purity is a crucial step not just for the final product but also during stability studies. It is essential to recognize that possible impurities might not just be ionic Zr^4+^ species but also chelated species. This is crucial when using *p*-NCS-Bz-DFO/DFO* since the thiourea bond shows a higher radiolysis sensitivity, especially in Cl^−^-containing buffers (Fig. 8). Decomposition species of ^89^Zr[Zr]-DFO-Bz-*p*-NCS (e.g., hydrolyzed or cleaved-off compounds) are lipophilic, which can lead to overestimations of the radiochemical purity, especially when common iTLC systems (solid phase: glass microfiber chromatography paper) are used as they poorly migrate with the mobile phase (Fig. 8). Therefore, determining the radiochemical purity with iTLC is recommended only for ^89^Zr[Zr]-DFO-*N*-suc-mAbs and precautions have to be taken when used for ^89^Zr[Zr]-DFO-Bz-*p*-NCS. SE-HPLC can be used for all radioconjugates if the recovery from the HPLC system is quantitative. An alternative method, using spin-filter analysis, is more recently reported (Vugts et al. 2017). But, one should continuously optimize the buffer for determining the radiochemical purity when using iTLC or spin filter since the outcome is also antibody-dependent and concentration-dependent. For all these methods, one has to perform proper controls not just with 89Zr-oxalate but also with other reference materials that reflect possibly impurities (e.g., hydrolyzed [^89^Zr]Zr-DFO-Bz-*p*-NCS in the presence of the mAb).

### Radiochemical yield

The radiochemical yield is not a direct parameter important for clinical applications. Nevertheless, consistent results should be expected when using standard procedures, and a radiochemical yield of ≥ 75% is generally accepted. If a lower radiochemical yield is obtained, there is a larger chance that the radiochemical purity is low. During radiolabeling, the radiochemical conversion can be monitored using iTLC or spin filter in the same manner as the radiochemical purity can be determined. Again, caution must be paid to choose the correct analysis method, depending on the bifunctional chelator used. To determine the radiochemical yield, the radioactivity of the reaction mixture is compared to the isolated product after size exclusion purification, which also takes additional losses (e.g., residues in the reaction vial) into account. This is also multiplied by the radiochemical purity as determined by SE-HPLC, spin-filter, or iTLC:

$$Radiochemical\, yield={Activity}_{product}*radiochemical\, purity/{Activity}_{reaction}$$

### Chemical purity

Determining chemical purity differs from small molecule radiotracers since no RP-HPLC analysis is done by default. For research purposes, it is usually assumed that the typical impurities (e.g., reaction buffer components, reactants, non-bound radioactivity, and solvent residues) are removed via the most common size exclusion purification methods (gravity columns, spin columns, or spin filtration).

When setting up clinical productions for patients, an evaluation has to be done for all potential impurities. One way to prove that the purification method is efficient in removing the reactants in the radiolabeling is by performing mock runs using the standard solutions of the impurities and purifying as is done with the radiolabeling mixtures, using water as eluent to enable a spectrophotometric analysis of the different reagents. Furthermore, LD50 values and other regulations (e.g., the use of FDA-approved drugs like Desferal) can be employed to determine whether the detected amounts raise a concern with regard to safety.

### Antibody integrity

As mentioned in Sect. "p-NCS-Bz-DFO and p-NCS-Bz-DFO* conjugation of antibodies", similar to the conjugation with the bifunctional chelators, the reaction conditions of the radiolabeling can result in precipitates and the formation of soluble non-covalent aggregates due to the build-up of unwanted non-covalent quaternary structures between multiple antibody molecules. Therefore, it is crucial to determine the antibody integrity via SE-HPLC and perform a visual check for cloudiness. SDS-PAGE is not suitable since SDS can dissociate unwanted build-up interactions between antibodies, hence giving false-positive results.

### Specific activity and antibody concentration

While for small-molecule tracers, the molar activity (amount of radioactivity (Bq)/moles of compound (mol)) or specific activity (amount of radioactivity (Bq)/gram of compound) is considered to be a vital value to determine, for radiolabeled antibodies, the perspective is slightly different.

Usually, a certain amount of antibody/kg body weight is required to prevent ‘antigen-sink,’ the target-mediated drug disposition with non-linear PK, or to saturate unspecific uptake in organs like the liver (Mohr et al. 2024). Therefore, after the radiolabeling with subsequent determination of antibody concentration via radio SE-HPLC, an amount of antibody is commonly added for multiple reasons: to prevent said ‘antigen-sink’ effect and to ensure a consistent specific activity (mass-dose consistency). Additionally, as discussed before (see formulation and final filtration), higher amounts of protein in the storage vial can reduce the percentage of radioactivity lost in the storage vial.

### Sterility, filter integrity, and endotoxin content

For clinical and preclinical preparations, it is mandatory to ensure sterility and low endotoxin content, which starts by always using sterile filtered solutions in the chelator conjugation and radiolabeling. Buffers that cannot be stored for an extended period (e.g., sucrose-containing buffers) have to be freshly prepared. For preclinical applications, radiolabeling is often not performed under sterile conditions. Hence, an additional sterile filtration with a 0.22 micron syringe filter before injection or storage is recommended.

For clinical productions, the production is performed in a GMP conform environment, and an additional sterile filtration of the final radiolabeled product in a grade A isolator is performed. The disposable filter used for this step has to be tested for integrity, usually via a pressure-hold test, where the filter has to withstand a certain amount of applied pressure before starting leakage. The sterility of the final product can be difficult to assess immediately since, most often, the radionuclide decay is needed to allow analysis at certified microbiology labs, which can lead to false positives or false negatives. Furthermore, sterile testing is never completed before injection due to the half-life of ^89^Zr and shelf-life of ^89^Zr-labeled antibodies, making it clear why following GMP regulations throughout the process is crucial. If sterility testing with the radioactive sample is impossible, mediafills and evaluating the sterile filtration process can be an alternative. Endotoxin content of the radiolabeled product can be determined via commercially available bacterial endotoxin tests (BET).

### Radioimmunoreactive fraction determination

The binding affinity of the radioimmunoconjugate to the corresponding antigen(s) can be determined using the traditional assay methods (e.g., ELISA, Biacore, Flow cytometry) that do not require a radioactive readout. However, these methods may not provide an accurate estimation of the binding affinity of the fraction of the radiolabeled antibody in the sample and may not always be practical from a radiation-handling standpoint. In those instances, binding assays designed to elucidate the binding activity of the radiolabeled antibody fraction in the sample should be utilized. These assays are often termed immunoreactivity determination or immunoreactive fraction (IRF) determination and involve in-vitro mixing of known amounts of radiolabeled antibody with excess antigen, applying a separation process that effectively removes unbound radiolabeled antibody from the sample containing the antigen and using radioactivity measurements to determine the fraction of radioactivity (representing the antibody) that is bound to the antigen. In the preclinical and early clinical setting, the most commonly utilized IRF assay, initially reported by Lindmo et al., is based on using the antigen in the form of cells having a high and stable expression of the target receptor or protein (Lindmo et al. 1984). However, using cells as antigen source (live or, preferably, fixated) might introduce variability and is sometimes difficult to validate as an analytical method. For these reasons, IRF assays involving stationary antigens coupled to either his-tag or biotinylated linkers capable of binding to magnetic beads or ELISA-type assays with a radioactive measurement of the % bound antibody are preferred in clinical production (Sharma et al. 2019). Accetapble maximum binding values, which can be seemingly low, differ depending on antibody and antigen combination from a perspective of kinetics (equilibrium) and affinity but also the time span of the assay (slower assays that could result in higher %bound antibody are not always applicable).

## Conclusion

The favorable PET imaging characteristics of ^89^Zr, combined with the excellent availability of the radionuclide due to its relatively simple production and purification processes, have led to widespread use of ^89^Zr following the development of suitable bifunctional chelators for labeling antibodies. The combination of antibodies and ^89^Zr, known as ^89^Zr-immuno-PET, has become a cornerstone in drug development in recent years, particularly for drug development and patient selection (Dongen et al. 2021). Despite the mature state of ^89^Zr-immuno-PET, developments in chelator conjugation and radiolabeling procedures, application on novel compound classes, and improved PET scanner technology and quantification methods continue to reshape its landscape.

The introduction of total body PET-CT scanners, characterized by significantly increased sensitivity compared to standard PET-CT scanners, has led to a notable decrease in the radioactivity required for the acquisition of high-quality scans (Dongen et al. 2021; Berg et al. 2020). This advancement enables repeated patient scans and provides safer conditions for healthy control groups in clinical trials. Also, it further extends the applicability of ^89^Zr-immuno-PET to targets with low abundance that would otherwise necessitate disproportionate amounts of injected radioactivity (Stergiou et al. 2023).

The utilization of ^89^Zr extends beyond antibodies to encompass various biologics, including peptides, affibodies, nanobodies, and antibody fragments (Ducharme et al. 2023; Ghosh et al. 2023; Bauer et al. 2022; Alizadeh et al. 2021; Xu et al. 2020). Additionally, other compound classes have been investigated with ^89^Zr, such as nanoparticles, micelles, living cells, and even microplastics (Friberger et al. 2023; Delaney et al. 2023; Zheng et al. 2022; Rijcken et al. 2022; Miedema et al. 2022). This diversity underscores the versatility of ^89^Zr, indicating its pivotal role in advancing different facets of life science.

In addition to the standard manual step-by-step operating procedures and protocols upon which this review is based, complementary work was conducted with automated synthesis modules. These modules can enhance reproducibility while minimizing radiation doses for the operator, which will have significant implications when large-scale routine productions are implemented (Wichmann et al. 2023; Poot et al. 2019). Furthermore, the standard procedures discussed in this review utilize random conjugations of amines on lysines, hence leading to heterogenous products and potentially hampering binding affinity and specificity (Rodriguez et al. 2022; Sadiki et al. 2020). Several recent publications have indicated the advantages of site-specific approaches for conjugation and subsequent ^89^Zr-labeling, but such advantages have to be assessed for each individual drug product (Rodriguez et al. 2023; Adumeau et al. 2022; Vivier et al. 2020; Kristensen et al. 2019; Yeh et al. 2024). Additionally, the homogenous products from these methods could potentially lower the burden for FDA approval. Notably, commercial kits are already available to facilitate the implementation of these site-specific strategies. A final consideration for future chelator conjugation and labeling procedures is the constant discovery of novel bifunctional and trifunctional (e.g., probes for dual-modality PET and optical imaging) chelator constructs (Feiner et al. 2021a; Adumeau et al. 2022; Guillou et al. 2022). Considering the relatively minor chemical difference between Fe–*N*-suc-DFO-TFP ester and *p*-NCS-Bz-DFO/DFO*, the resulting differences in nearly all aspects of the procedures for chelator conjugation and quality controls are evident. Therefore, it is crucial to scrutinize the guidelines outlined in this review and re-adjust procedures accordingly.

## Data Availability

Not applicable.

## Abbreviations

aCSF: Artificial cerebrospinal fluid
ALARA: As low as reasonable possible
BET: Bacterial endotoxin test
Biacore: Biomolecular interaction analyzing system
Bz: Phenyl
CT: Computed tomography scan
Df: Desferal
DFO: Desferrioxamine
DFO*: DFOstar
DFOA: Deferoxamine
DFOB: Desferrioxamine B
DMSO: Dimethyl sulfoxide
DOTA: Dodecane tetraacetic acid
EDTA: Ethylenediaminetetraacetic acid
ELISA: Enzyme-linked immunosorbent assay
EOI: End-of-irradiation
eq.: Equivalent
EU/mL: Endotoxin units per milliliter
HEPES: 4-(2-Hydroxyethyl)-1-piperazineethanesulfonic acid
HOPO: Hydroxypyridinone
ICP-MS: Inductively coupled plasma-mass spectrometry
IRF: Immunoreactive fraction
iTLC: Instant thin-layer chromatography
LC-ESI-MS: HPLC-electrospray ionization mass spectrometry
LD50: Median lethal dose
LLOD: Lower limit of detection
LLOQ: Lower limit of quantification
LoD: Limit of detection
LoQ: Limit of quantification
mAb: Monoclonal antibody
mal: Maleimid
MALDI-TOF: Matrix-assisted laser desorption/ionisation time-of-flight mass spectrometry
PBS: Phosphate buffered saline
PD: Preparative desalting size exclusion column
PET: Positron emission tomography
QC: Quality control
SATA: *N*-Succinimidyl-S-acetylthioacetate
SDS-PAGE: Sodium dodecyl-sulfate polyacrylamide gel electrophoresis
SE-HPLC: Size-exclusion high-performance liquid chromatography
Sq: Squaramide
Suc: Succinyl
Tween: Polysorbate
UV-Vis: Ultraviolet–visible
